# Pitavastatin Induces Cancer Cell Apoptosis by Blocking Autophagy Flux

**DOI:** 10.3389/fphar.2022.854506

**Published:** 2022-03-21

**Authors:** Nirmala Tilija Pun, Naeun Lee, Sang-Hoon Song, Chul-Ho Jeong

**Affiliations:** ^1^ College of Pharmacy, Keimyung University, Daegu, South Korea; ^2^ Boston Children’s Hospital, Boston, MA, United States

**Keywords:** pitavastatin, autophagy flux blockade, apoptosis, ER stress, FOXO3a

## Abstract

Statins, a class of lipid-lowering drugs, are used in drug repositioning for treatment of human cancer. However, the molecular mechanisms underlying statin-induced cancer cell death and autophagy are not clearly defined. In the present study, we showed that pitavastatin could increase apoptosis in a FOXO3a-dependent manner in the oral cancer cell line, SCC15, and the colon cancer cell line, SW480, along with the blockade of autophagy flux. The inhibition of autophagy by silencing the LC3B gene reduced apoptosis, while blockade of autophagy flux using its inhibitor, Bafilomycin A1, further induced apoptosis upon pitavastatin treatment, which suggested that autophagy flux blockage was the cause of apoptosis by pitavastatin. Further, the FOXO3a protein accumulated due to the blockade of autophagy flux which in turn was associated with the induction of ER stress by transcriptional upregulation of PERK-CHOP pathway, subsequently causing apoptosis due to pitavastatin treatment. Taken together, pitavastatin-mediated blockade of autophagy flux caused an accumulation of FOXO3a protein, thereby leading to the induction of PERK, ultimately causing CHOP-mediated apoptosis in cancer cells. Thus, the present study highlighted the additional molecular mechanism underlying the role of autophagy flux blockade in inducing ER stress, eventually leading to apoptosis by pitavastatin.

## Introduction

Globally, cancer is the leading cause of death, contributing to approximately 19.3 million new cases and 10 million deaths in 2020 (Sung et al., 2021). Due to the progressive increase in the new cases and deaths due to cancer, the discovery of new drugs or alternative ways for cure is important. Drug repurposing is an alternative strategy to use old drugs for new therapeutic purposes; this skips the necessity of preclinical and clinical studies, thereby reducing the discovery time and investment costs (Zhang et al., 2020). This approach has been especially applied in primary therapy for rare tumors lacking effective treatments and/or to develop secondary treatments for recurrent diseases (Wurth et al., 2016).

Statins, inhibitors of hydroxymethylglutaryl-coenzyme A (HMG-COA) reductase, an enzyme that produces cholesterol, are commonly prescribed medications for the treatment of hyperlipidemia and atherosclerosis, owing to their lipid and cholesterol-lowering effects (Bardou et al., 2010). Accumulation of lipid and cholesterol are the prominent characteristics of tumors that play a role in carcinogenesis and malignant development of cancers (Sharma and Agnihotri, 2019; Li et al., 2020). Due to the lipid and cholesterol-lowering properties of statin, it has been repurposed in recent years as an anti-cancer drug for breast, colon, and other cancer types (Bardou et al., 2010; Borgquist et al., 2018; Joharatnam-Hogan et al., 2021). For example, a clinical study for hyperlipidemic prostate cancer patients in Taiwan reports that statins decrease the mortality rate in these patients (Chen et al., 2018). In addition, statins exhibit anti-cancer actions through other mechanisms unrelated to cholesterol production. Among statins, pitavastatin exhibits the most potent effect in decreasing cancer cell viability in breast cancer and glioblastoma (Jiang et al., 2014a; Jiang et al., 2014b). However, the complete molecular mechanism underlying the anti-proliferative effects of pitavastatin in cancer cells remains unclear.

Autophagy is a self-degradation process in which the intracellular components are degraded by sequestration into a double-membrane structure (autophagosome), followed by its fusion with lysosomes, eventually leading to lysosomal-mediated degradation. The sequestration and enclosure of cytoplasmic components into the double-membrane structure is mediated by protein complex, namely, the Class III PI3K complex (including Beclin1, VPS34, and Atg14); conjugation of two protein complexes, ATG5-ATG16 and LC3II (Tan et al., 2017; Devis-Jauregui et al., 2020), and finally, the fusion of the autophagosome with lysosomes through the action of three protein complexes, SNAP receptors, tethering factors, and Rab GTPases (Tilija Pun et al., 2020). Autophagy is a well-known process in the long-term survival of cancer cells. However, recent studies focus on the converse role of autophagy in inducing cell death (autophagic cell death) and apoptosis (Tilija Pun et al., 2020; Park et al., 2021). For instance, autophagy induces cell death even upon inhibition of apoptotic pathways through double knockouts of BAX and BAK, or in presence of caspase inhibitors (Yonekawa and Thorburn, 2013). Further, autophagy triggers cathepsin D (CTSD) maturation and localization inside the midgut cells to activate caspase-3 and promotes apoptosis (Di et al., 2020). Overexpression of autophagy-inducing protein, Beclin-1, decreases oral squamous cancer cell proliferation, migration, and invasion (Weng et al., 2014). Furthermore, rapamycin, an autophagy inducer, decreases tongue squamous cancer cell proliferation, invasion, and wound healing (Wang et al., 2013). The conversion of cytoprotective autophagy to cytostatic autophagy mediates cytotoxicity in non-small cell lung cancer (Sharma et al., 2014). Further, cytostatic autophagy induced by the combination ivermectin and adiponectin causes cell death in breast cancer (Dou et al., 2016; Chung et al., 2017). These results suggest that autophagy processes are not only involved in cancer cell growth but also in its inhibition. In recent years, cell death mediated by autophagy is shown to be associated with the blockade of autophagy flux. For example, IMB-6G-induced apoptosis in pancreatic cancer cells is linked to the autophagy flux blockade or lysosomal dysfunction (Liu et al., 2017). A recent review suggests that many anti-cancer drugs can inhibit the autophagy flux to induce cell death or apoptosis (Tilija Pun et al., 2020). Although pitavastatin induces cytotoxicity in melanoma cells along with the induction of autophagy, ultimately leading to cell death (Al-Qatati and Aliwaini, 2017), the complete molecular mechanism underlying the effects of pitavastatin on autophagy-mediated cell death and apoptosis in cancer cells are unclear.

Endoplasmic reticulum (ER), a membranous network is a major synthesis site for almost all secretary and integral membrane proteins; its function includes protein folding, calcium buffering, and lipid and carbohydrate metabolism (Rashid et al., 2015; Song et al., 2018). Intrinsic perturbations in disease conditions, such as in cancer, neurodegenerative diseases, and diabetes, and extrinsic perturbations such as microenvironmental stressors (nutrient deprivation), exposure to ER stressors (tunicamycin), temperature, and reactive oxygen species (ROS) can disrupt ER homeostasis, thereby inducing stress (Almanza et al., 2019). As a result, unfolded protein response (UPR) is activated by reducing protein synthesis, clearing the unfolded proteins, and increasing the capacity of the ER to fold proteins, to cope with the stress. The three major sensors that control the UPR system include inositol requiring enzyme 1 (IRE1), protein kinase RNA-activated (PKR)-like ER kinase (PERK), and activating transcription factor 6 (ATF6), eventually leading to C/EBP homologous protein (CHOP) induction (Almanza et al., 2019). However, persistent ER stress causes cell death or apoptosis. Accumulating evidence suggests that ER stress induces cellular dysfunction and cell death in several diseases (Sano and Reed, 2013). ER stress not only induces autophagy but also inhibits its function by inhibiting the lysosomal activity (Rashid et al., 2015). Interestingly, the elevated level of ER stress disrupts autophagy flux that subsequently leads to apoptosis in liver cells from patients of non-alcoholic fatty liver disease (NAFLD) (González-Rodríguez et al., 2014). Although ER stress and autophagy-inducing effects of statin are well known (Mollazadeh et al., 2018), the roles of statin-mediated autophagy in ER stress induction or vice versa are not well understood.

Therefore, to bridge the knowledge gap between statin-mediated autophagy and the subsequent role of autophagy in cell death, the present study aimed to investigate the role of pitavastatin in autophagy induction, autophagy flux regulation, ER stress induction, and cell death in cancer cell lines. We demonstrated that pitavastatin-mediated blockade of autophagy flux caused the accumulation of FOXO3a protein, thereby leading to the induction of ER stress-mediated apoptosis in oral and colon cancer cells.

## Materials and Methods

### Chemicals and Reagents

All the cell culture reagents were purchased from Hyclone Laboratories (South Logan, UT, United States). Pitavastatin calcium was purchased from HL Genomics (Gyeonggi-Do, Republic of Korea). Bafilomycin A1, 3-methyal-adenine (3-MA), lys05, 4′,6-diamidino-2-phe-nylindole (DAPI), dimethyl sulfoxide (DMSO), cycloheximide (CHX), PERK inhibitor (GSK2606414), and tunicamycin were obtained from Sigma-Aldrich (St. Louis, MO, United States). 4-Phenylbutyric acid (4PBA) and IRE1 inhibitor (MKC8866) were purchased from MCE Med Chem Express (Monmouth Junction, NJ 08852, United States). Rapamycin, primary antibodies against caspase9, caspase3, PARP, actin, lamin B1, LC3B, p62, STX17, LAMP1, cathepsin B, FOXO3a, GRP78, PERK, eIF2α, p-eIF2α (Ser51), ATF4, CHOP, IRE1α, XBP1s, and the secondary antibodies, were purchased from Cell Signaling Technology (Beverly, MA, United States).

### Cell Lines and Cell Culture

Human oral squamous cancer cell lines, SCC4 and SCC15, human colon cancer cell lines, HCT116, HT29, and SW480, were obtained from the American Type Culture Collection (ATCC). The SCC4 and SCC15 cells were cultured in Dulbecco’s modified Eagle’s medium/F12 (DMEM/F12 = 1:1) supplemented with 10% fetal bovine serum (FBS, Atlas Biologicals, Fort Collins, CO, United States) and antibiotics, 100 U/mL penicillin and 100 mg/ml streptomycin (HyClone Laboratories, Logan, UT, United States). The HCT116, HT29, and SW480 cells were cultured in RPMI-1640 medium supplemented with 10% fetal bovine serum (Atlas Biologicals) and antibiotics, 100 U/mL penicillin and 100 mg/ml streptomycin (HyClone). All cells were grown in a humidified atmosphere containing 5% CO_2_ at 37°C.

### Cell Viability Assay

Cell viability was measured using the CytoTox-Glo cytotoxicity assay kit (Promega Corporation, Madison, WI) according to the manufacturer’s protocol. Cell viability was calculated by subtracting the luminescent signal of dead cells from the luminescent signal of the total number of cells. Luminescence was detected using FLUOstar Omega (BMG Labtech, Germany).

### Transient Transfection With Small Interfering RNA

Double-stranded siRNAs against human LC3B (NM_022818.4), FOXO3a (NM_001455.3), STX17 (NM_017919.2), and CHOP (NM_001195053.1) were synthesized by Bioneer (Daejeon, Republic of Korea). Negative control siRNA constructs were obtained from Bioneer (Cat. No: SN-1003). The sequences for siRNA duplexes used in this study are as follows: LC3B: forward, 5′-CAU AAA GAC ACC ACU CAA A-3′; reverse, 5′-UUU GAG UGG UGU CUU UAU G-3′; STX17: forward, 5′- ACU UCU CUC UCC UAG UGA A-3′; reverse, 5′-UUC ACU AGG AGA GAG AAG U-3′; FOXO3a: forward, 5′-CUA UCA UAU GGC AUU CUU A-3′; reverse, 5′- UAA GAA UGC CAU AUG AUA G-3′, and CHOP: forward, 5′- CAG AAG UGG CUA CUG ACU A-3′; reverse, 5′-UAG UCA GUA GCC ACU UCU G-3′.

### Caspase-3/7 Activity Assay

Caspase-3/7 activity was assessed using the Caspase-Glo 3/7 assay kit (Promega Corporation, Madison, WI) according to the manufacturer’s instructions. Cells were seeded at a density of 2× 10^3^ cells/well in 96-well microplates and incubated overnight. After drug treatment, cells were incubated with the luminogenic substrate; caspase-3/7 activity was determined by measuring luminescence using a micro-plate reader. The signal was generated due to the cleavage of the luminogenic substrate, Z-DEVD-NH, in presence of caspase3/7 protease.

### Western Blot Analysis

Total cellular extracts were obtained using the radio-immunoprecipitation assay lysis buffer (RIPA) containing the Halt Protease Inhibitor Cocktail (Thermo Scientific). Next, 20–30 µg of total proteins were loaded onto 8–13% sodium dodecyl sulfate-polyacrylamide gel (SDS-PAGE), separated by electrophoresis, and transferred onto PVDF membranes (Millipore Corp., Bedford, MA, United States). Non-specific binding was blocked by incubating the membrane with 5% skim milk in TBS-T for 30 min. Next, the membrane was incubated overnight at 4°C with specific primary antibodies. After incubation with species-specific horseradish peroxidase-conjugated secondary antibodies, proteins were visualized using SuperSignal^®^ West Dura Extended Duration Substrate (Thermo Scientific, Waltham, MA) and developed on the LAS-3000 (Fuji, Japan) platform according to the manufacturer’s instructions.

### Flow Cytometry

Cell apoptosis was detected using the FITC Annexin V apoptosis detection kit with 7-AAD (Biolegend, San Diego, CA) as per the manufacturer’s protocol. Briefly, cells were seeded at a density of 1.2 × 10^5^ cells/well in 6-well plates and incubated overnight. After drug treatment, the cells were collected, washed twice with cold PBS and re-suspended in binding buffer. Next, 5 μL of FITC Annexin V and 7-AAD were added to 100 μL of the cell suspension and the cells were incubated for 15 min at RT in dark. The analysis was performed on a BD FACSVerse flow cytometer platform with BD FACSuite Software. The fraction of cell population in different quadrants was analyzed using quadrant statistics.

### Immunocytochemistry

Cells were plated onto submerged glass coverslips and seeded at 2 × 10^4^ cells/well in 6-well plates for 24 h. After drug treatment, cells were fixed with methanol for 2 min and permeabilized with 0.1% Triton X-100 in DPBS for 10 min at RT. Coverslips were blocked with 1% BSA in DPBS-T, and cells were incubated with antibodies against LC3B, overnight at 4°C. After washing with DPBS-T and incubating with the Alexa 488-conjugated secondary antibody for 90 min at RT in the dark, nuclei were counterstained with 0.2 μg/ml DAPI. Images were then obtained using a Carl Zeiss LSM 5 exciter confocal microscope (ZEISS, Germany).

### mCherry-GFP-LC3 Puncta Assay by Confocal Microscopy

Cells were plated at a density of 5 × 10^4^ cells/well in 6-well chamber slides and allowed to adhere overnight. Cells were then transfected with mCherry-GFP-LC3B plasmid (Addgene, Watertown, MA, United States) using the jetPEI^®^ transfection reagent (Polyplus Transfection, Illkirch, France). After drug treatment, cells were fixed with 4% formaldehyde at RT for 15 min, nuclei were counterstained with 0.2 μg/ml DAPI. Images were obtained using the Carl Zeiss LSM 5 exciter confocal microscope (ZEISS, Germany).

### Detection of Misfolded Proteins

Misfolded proteins in cells were detected using the ProteoStat Aggresome Detection Kit (Enzo Life Sciences, Farmingdale, N.Y. United States). Cells were plated onto submerged glass coverslips and seeded at 5 × 10^4^ cells/well in 6-well plates. After treatment, the slides were incubated with 4% formaldehyde at RT for 30 min and permeabilized on ice for 30 min with permeabilizing solution (0.5% Triton X−100, 3 mM EDTA, pH 8). Next, the cells were stained with a dual detection reagent containing Hoechst 33342 (nuclear stain) and ProteoStat aggresomal detection reagent and incubated for 30 min at RT. After removing excess buffer and placing the coverslips on the microscope slides, the stained cells were analyzed for aggresomal and nuclear signals using the Carl Zeiss LSM 5 exciter confocal microscope (ZEISS, Germany).

### Statistical Analyses

Data were analyzed using Student’s *t*-test or one/two-way ANOVA using GraphPad Prism 5. All data are presented as mean ± SD. *p-*value less than 0.05 (*p* < 0.05) was considered statistically significant.

## Results

### Pitavastatin Induces Apoptosis in SCC15 and SW480 Cells

**FIGURE 1:**
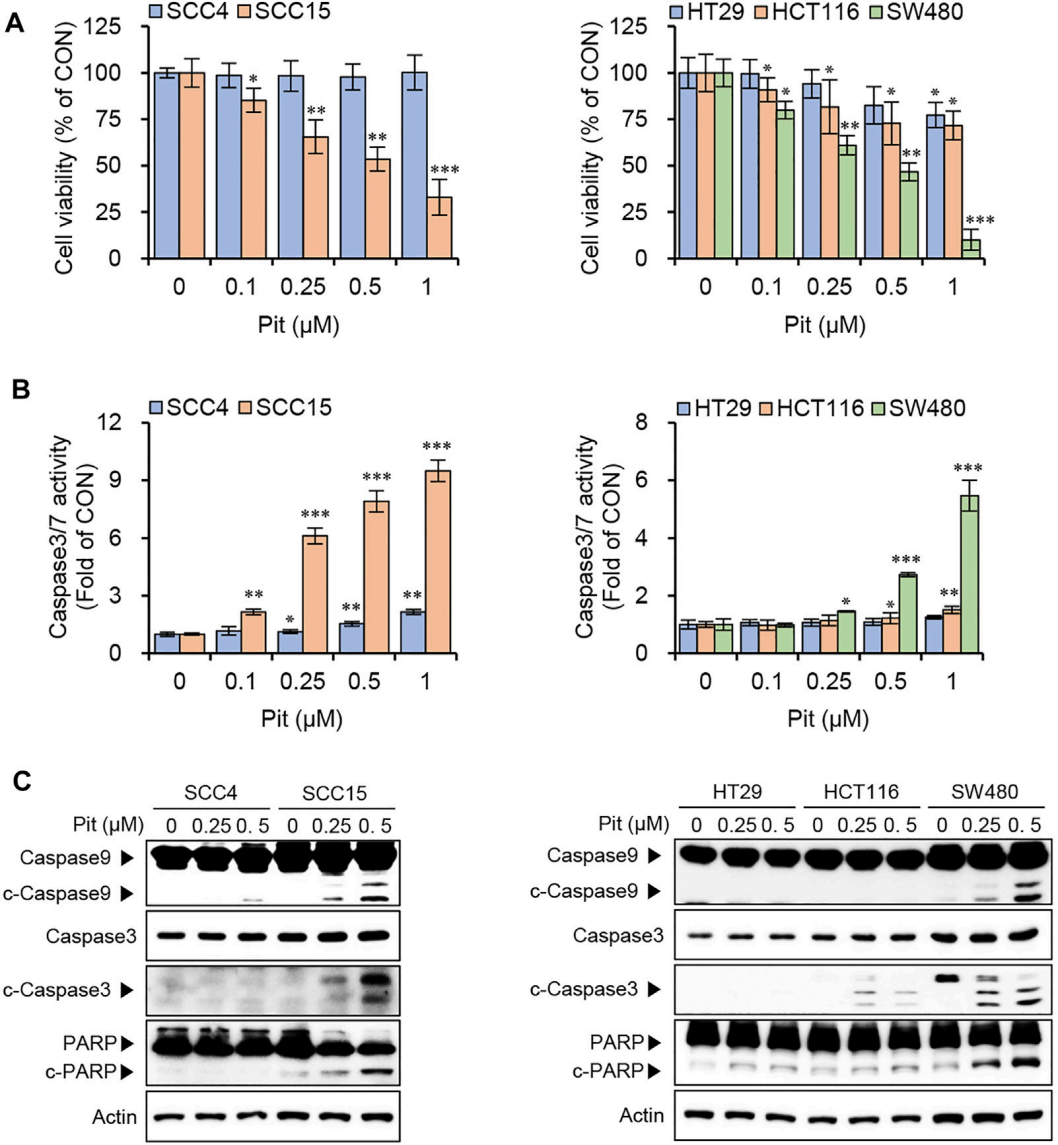
Pitavastatin induces apoptosis in SCC15 and SW480 cells. **(A)** Cells were seeded onto 96-well plates (1.5 × 10^3^ cells/well) and treated with various concentrations of pitavastatin for 48 h. Cell viabilities were measured using the CytoTox-Glo cytotoxicity assay kit. Statistical analysis was performed using the Student’s *t*-test. Error bars represent mean ± SD (*n* = 4). ^*^ *p* < 0.05; ^**^ *p* < 0.01; ^***^ *p* < 0.001. **(B)** Cells seeded at a density of 1.5 × 10^3^ cells/well were treated with pitavastatin for 48 h and caspase 3/7 activity was measured using the Caspase 3/7 Glo assay kit. Statistical analysis was performed using Student’s *t*-test. Error bars represent mean ± SD (*n* = 4). ^*^ *p* < 0.05; ^**^ *p* < 0.01; ^***^ *p* < 0.001. **(C)** Oral cancer cells (3 × 10^5^ cells/well) and colon cancer cells (4 × 10^5^ cells/well) seeded in 60 mm dish were treated with pitavastatin for 24 h and protein levels of caspase9, caspase3 and PARP were measured by western blot analyses; actin was used the internal loading control.

To evaluate the anti-cancer effects of pitavastatin on human oral cancer cells (SCC4 and SCC15) and colon cancer cells (HT29, HCT116, and SW480), the cell viability assay was performed. Treatment of cells with pitavastatin showed that cell viability was selectively and significantly reduced in oral cancer cells, SCC15 and colon cancer cells, SW480 among tested cancer cell lines (Figure 1A). This was accompanied by an increase in the caspase3/7 activity in both SCC15 and SW480 cells (Figure 1B). Additionally, the expressions of apoptotic markers, cleaved forms of caspase9, caspase3, and PARP increased significantly upon treatment with 0.25–0.5 μM pitavastatin in both the SCC15 and SW480 cells (Figure 1C) in a dose-dependent manner. These results suggest that pitavastatin could selectively decrease cell viability and induce apoptosis in SCC15 and SW480 cells.

### Autophagy is Involved in Pitavastatin-Induced Apoptosis in SCC15 and SW480 Cells

**FIGURE 2:**
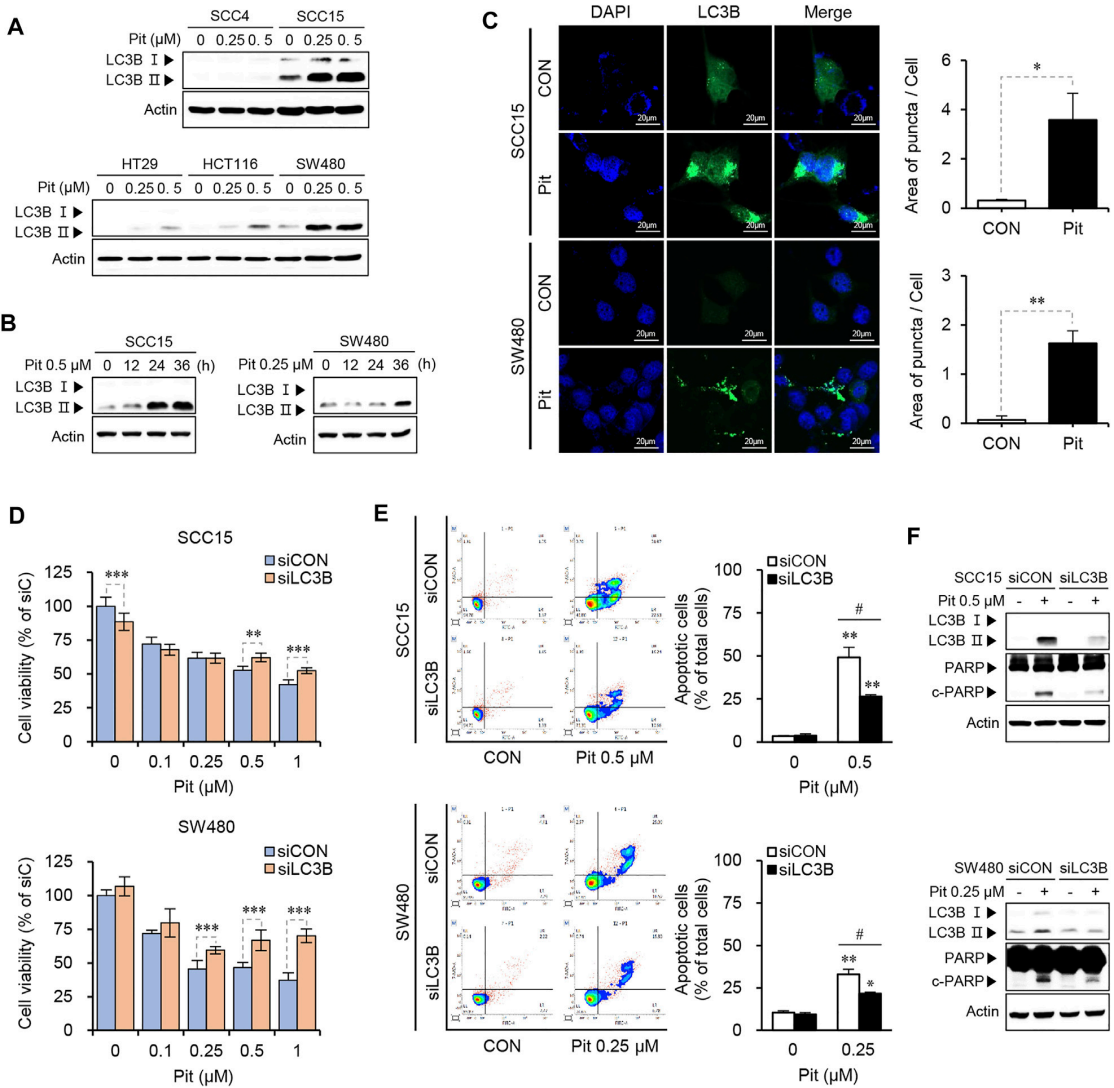
Pitavastatin induces autophagy-dependent apoptosis in SCC15 and SW480 cells. **(A,B)** Cells were treated with pitavastatin as indicated. The expressions of LC3B-I and LC3B- II proteins were detected using western blot analysis. Actin was used as the loading control. **(C)** SCC15 and SW480 cells seeded on sterile coverslips were transfected with GFP-LC3 plasmid for 24 h, followed by treatment with or without pitavastatin for 36 h, and the formation of autophagosomes (green puncta) was analyzed by confocal microscopy. The histograms show the quantification of GFP puncta, quantitated using the ImageJ software. Statistical analysis was performed using the Student’s *t*-test. Error bars represent mean ± SD (*n* = 3). ^*^ *p* < 0.05; ^**^ *p* < 0.01. **(D)** SCC15 and SW480 cells plated on 96-well plates were transfected with LC3B siRNA in the presence or absence of the indicated concentrations of pitavastatin for 48 h. Cell viability was then measured using the CytoTox-Glo cytotoxicity assay. Statistical analysis was performed using two-way ANOVA. Error bars represent mean ± SD (*n* = 4). ^**^ *p* < 0.01; ^***^ *p* < 0.001. **(E)** Flow cytometry analysis was performed to evaluate the effect of siLC3B on the apoptosis of SCC15 and SW480 cells. Statistical analysis was performed using the Student’s *t*-test. Error bars represent mean ± SD (*n* = 3). ^*^ *p* < 0.05; ^**^ *p* < 0.01; ^#^ *p* < 0.05. **(F)** After transfection with LC3B siRNA, cells were further incubated for 24 h in the presence or absence of pitavastatin. The protein levels were measured by western blot analyses.

Several studies report that statin treatment induces autophagy and either induces or inhibits cell death/apoptosis, depending on cancer cell types and/or statin type, and co-treatment options (Hwang et al., 2015; Yang et al., 2017). As the role of autophagy in cancer cell death and apoptosis remains controversial, the effects of pitavastatin on the autophagy process were investigated in our study. Our data showed that treatment with pitavastatin could induce the expression of LC3B-II, an indicator of autophagy, with the highest level observed in SCC15 and SW480 cells in both dose- and time-dependent manners (Figures 2A,B). Induction of autophagy by pitavastatin was also confirmed by autophagosome formation indicated by the green puncta in LC3B-transfected SCC15 and SW480 cells, which suggested the effects of pitavastatin on autophagy induction in both these cancer cell lines (Figure 2C). Next, the role of autophagy in pitavastatin-induced apoptosis in SCC15 and SW480 cells was investigated by gene silencing of LC3B. The data showed that the decreased cell viability upon pitavastatin treatment was significantly rescued by LC3B gene silencing in SCC15 and SW480 cells (Figure 2D). Moreover, gene silencing of LC3B alleviated the pitavastatin-induced apoptotic cell death (Figure 2E) and PARP cleavage (Figure 2F). These results suggested that the anti-cancer effects of pitavastatin were mediated by the autophagy processes in SCC15 and SW480 cells.

### Pitavastatin Induces Apoptosis by Blockade of Autophagy Flux in SCC15 and SW480 Cells

**FIGURE 3:**
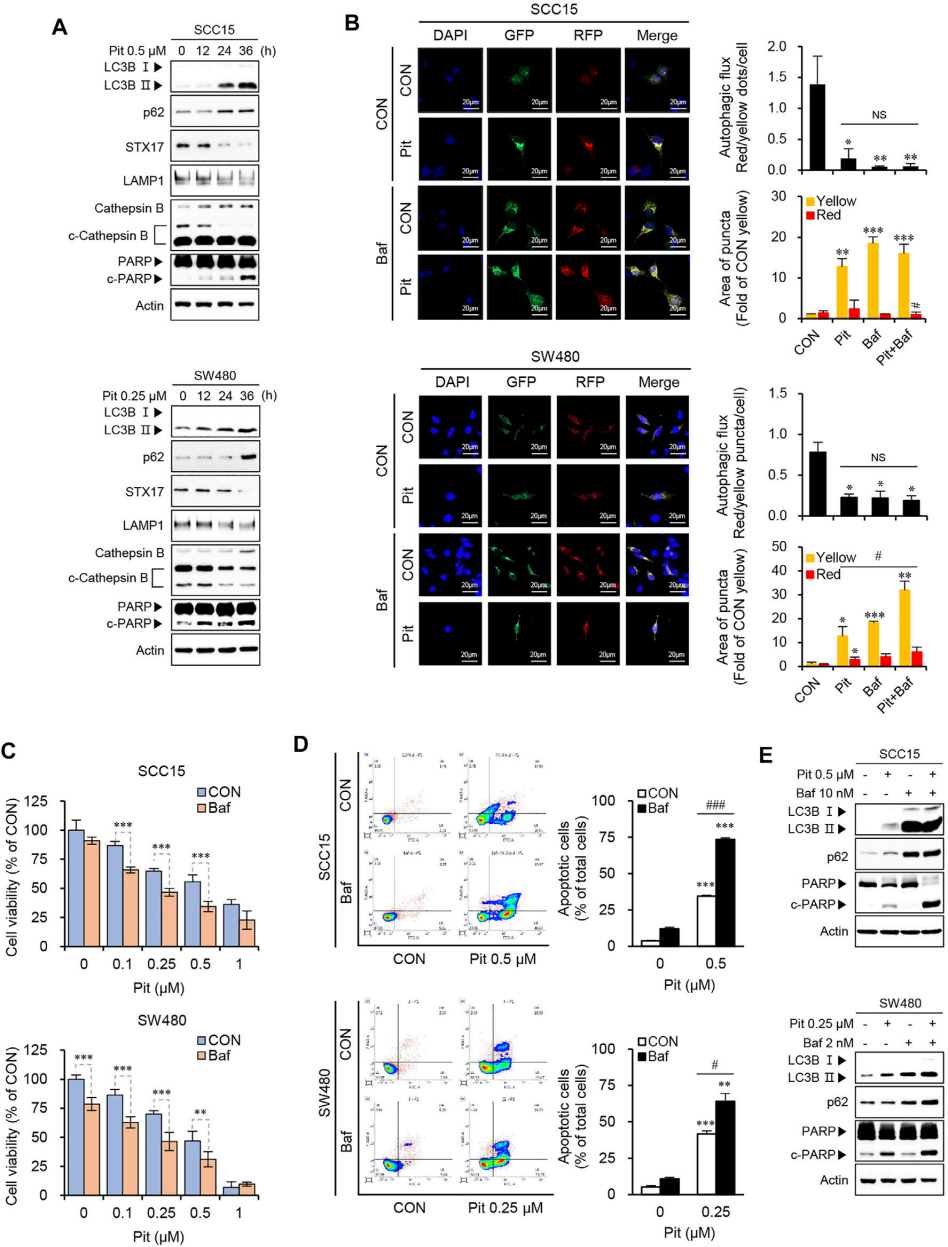
Pitavastatin induces apoptosis through the blockade of autophagy flux. **(A)** Cells were treated with 0.5 µM of pitavastatin as indicated and the protein expressions were measured by western blot analyses. **(B)** After transfection of cells with mCherry-GFP-LC3 plasmid, cells were treated with pitavastatin or Bafilomycin A1 for 36 h. The formation of autophagosomes (yellow puncta), and autolysosomes (only red puncta) were analyzed by confocal microscopy. The histograms show the quantification of yellow and red puncta; quantitation was performed using the ImageJ software. Statistical analysis was performed using the Student’s *t*-test. Error bars represent mean ± SD (*n* = 3). ^*^ *p* < 0.05; ^**^ *p* < 0.01; ^***^ *p* < 0.001; ^#^ *p* < 0.05. **(C)** Cells were treated with pitavastatin and Bafilomycin A1 together for 48 h, and the cell viability was measured by using the CytoTox-Glo cytotoxicity assay. Statistical analysis was performed by two-way ANOVA. Error bars represent mean ± SD (*n* = 4). ^**^ *p* < 0.01; ^***^ *p* < 0.001. **(D)** After treatment with pitavastatin and/or Bafilomycin A1 for 48 h, the rates of apoptosis were measured by flow cytometric analysis with Annexin V staining (left panel). The quantification of apoptotic cells is shown by the graph (right panel). Statistical analysis was performed using Student’s *t*-test. Error bars represent mean ± SD (*n* = 3). ^**^ *p* < 0.01; ^***^ *p* < 0.001; ^#^ *p* < 0.05; ^###^ *p* < 0.001. **(E)** Cells were treated with pitavastatin and/or Bafilomycin A1 for 24 h and the indicated proteins were detected by western blot analysis.

The fusion of autophagosomes and lysosomes is important for autophagy flux and inhibition of this process impairs autophagic degradation (Mizushima et al., 2010). Therefore, we evaluated the autophagy flux to understand whether pitavastatin affected the entire process of autophagy or only its late stages. The expressions of p62, syntaxin 17 (STX17), LAMP1, and cathepsin B were measured using western blot analysis. As shown in Figure 3A, pitavastatin treatment increased the expression of p62 along with the increase in LC3B-II levels, which suggested dysfunction in the autophagy flux. Further, STX17 expression decreased, which indicated the failure of autophagosome fusion with endosome/lysosome (Itakura et al., 2012). Decreased LAMP1 protein expression, and cathepsin B activation indicated the dysfunction in lysosomes in a time-dependent manner in both the SCC15 and SW480 cells. Taken together, these results suggested that pitavastatin blocked autophagy flux by suppression of fusion proteins and inhibition of lysosomal function. The inhibition of autophagy flux by pitavastatin was further confirmed by measuring the occurrences of autophagosomes and autophagosome-lysosomal fusions using overexpressing mCherry-GFP-LC3, a tandem fluorescent indicator construct. Since the green fluorescence of the fusion protein is sensitive to the acidic environment in lysosomes, it is quickly quenched in the lysosomes and only the red fluorescence is detected in the autolysosomes. Our data showed that pitavastatin increased both green and red fluorescence signals (yellow, GFP^+^mCherry^+^ puncta) which was further enhanced by bafilomycin A1 pretreatment in both SCC15 and SW480 cells. This suggested that pitavastatin blocked the autophagy flux (Figure 3B). Consistently, the decrease in cell viability upon pitavastatin treatment was further exacerbated by bafilomycin A1 pretreatment, which suggested the role of autophagy flux blockade in suppression of cell viability by pitavastatin (Figure 3C). Moreover, based on the flow cytometry data, as shown in Figure 3D, the combined treatment with pitavastatin and bafilomycin A1 significantly increased early and late apoptosis in SCC15 and SW480 cells as compared to treatment with either of the drugs singly. Pitavastatin-induced expressions of LC3B-II, p62, and the cleavage of PARP protein also increased dramatically upon bafilomycin A1 pretreatment, which suggested that the autophagy flux was disrupted, thereby inducing apoptosis in SCC15 and SW480 cells (Figure 3E). Taken together, these results demonstrated that autophagy flux blockade by pitavastatin contributed to apoptosis in SCC15 and SW480 cells.

### Pitavastatin-Mediated Autophagy Flux Blockade Stabilizes FOXO3a Protein

**FIGURE 4:**
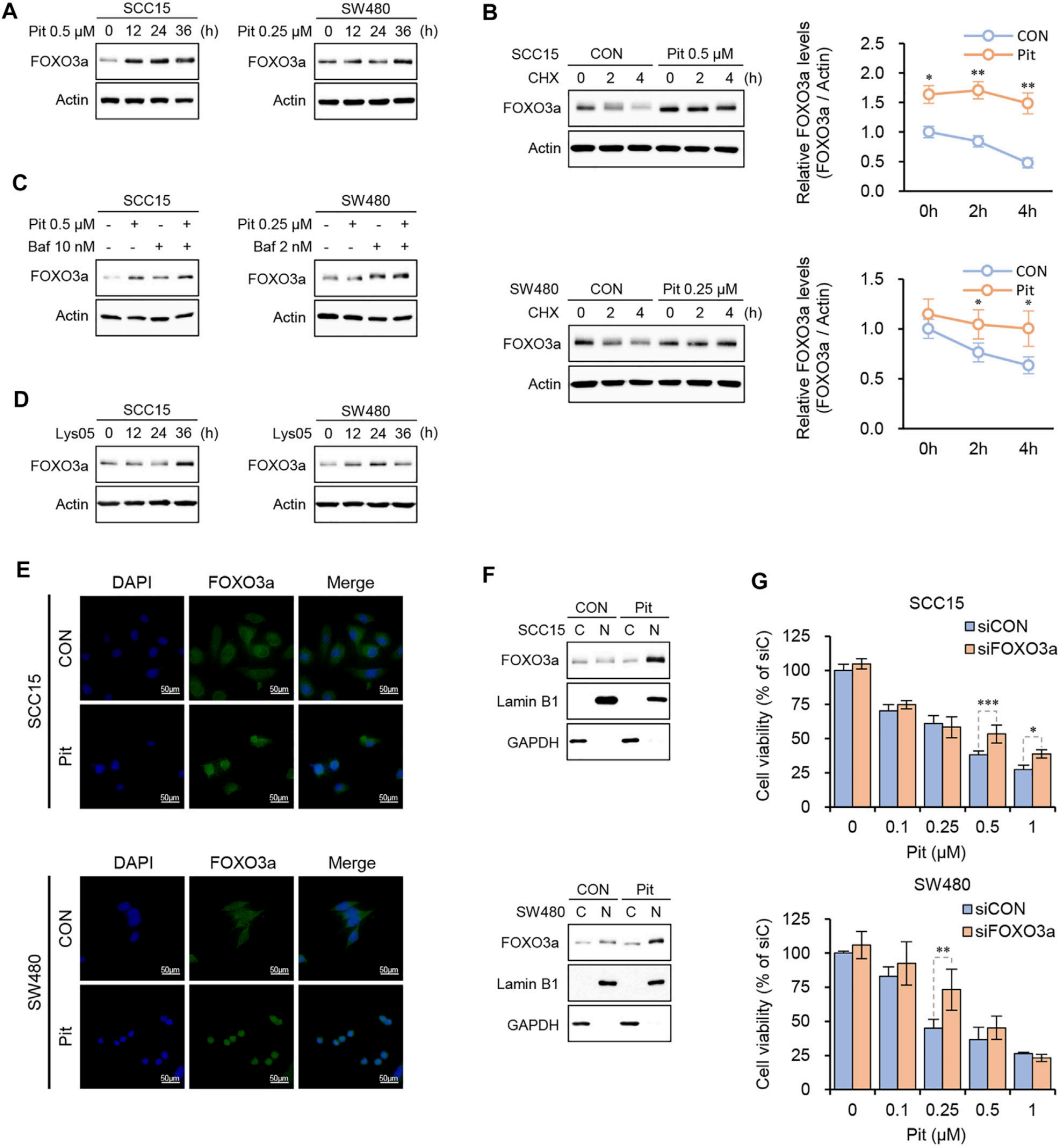
Pitavastatin increased the stability of FOXO3a protein. **(A)** Cells were treated with pitavastatin for the indicated times and the protein levels of FOXO3a were measured by western blot analyses. **(B)** Cells were treated with pitavastatin in presence or absence of CHX (10 μg/ml) to measure the degradation dynamics of FOXO3a by pitavastatin. ImageJ software was used for quantitation of FOXO3a protein levels. Statistical analysis was performed using two-way ANOVA. Error bars represent mean ± SD (*n* = 3). ^*^ *p* < 0.05; ^**^ *p* < 0.01. **(C,D)** Cells were treated with pitavastatin in the presence or absence of Bafilomycin A1 **(C)** or treated with Lys05 (3 µM) **(D)** for 24 h, and the protein level of FOXO3a was measured by western blot analyses. **(E)** SCC15 and SW480 cells seeded on sterile coverslips were treated with pitavastatin for 24 h. Cells were then fixed with methanol, followed by incubation with fluorescently tagged rabbit polyclonal FOXO3a antibody and stained with Alexa Fluor 488 (green)-labelled anti-rabbit antisera and DAPI. FOXO3a signals were then visualized by confocal microscopy. **(F)** After treatment of SCC15 and SW480 cells with pitavastatin for 24 h, the nuclear translocation of FOXO3a protein was measured by western blot analyses with GAPDH and Lamin B1 as the cytoplasmic and nuclear loading controls, respectively. **(G)** Cells plated on 96-well plates were transfected with FOXO3a siRNA in the presence or absence of the indicated concentrations of pitavastatin and incubated for 48 h. Cell viability was then measured using the CytoTox-Glo cytotoxicity assay. Statistical analysis was performed by two-way ANOVA. Error bars represent mean ± SD (*n* = 4). ^*^ *p* < 0.05; ^**^ *p* < 0.01; ^***^ *p* < 0.001.

In our previous study, we found that pitavastatin induces apoptosis through nuclear translocation of FOXO3a leading to PUMA induction in SCC15 cells. (Lee et al., 2020). Here, we checked the relationship between FOXO3a and autophagy flux regulation by pitavastatin. As shown in Figure 4A, pitavastatin increased FOXO3a protein expression in a time-dependent manner in both SCC15 and SW480 cells. Next, the protein synthesis inhibitor, cycloheximide (CHX), was used to confirm whether the accumulation of FOXO3a protein was due to the protein synthesis process. Interestingly, co-treatment of cells with CHX and pitavastatin did not decrease and the increased protein level of FOXO3a was sustained as compared to cells treated with CHX only, which indicated that pitavastatin stabilized the FOXO3a protein (Figure 4B). Our data also showed that the expression of FOXO3a protein by pitavastatin was further enhanced upon pretreatment with Bafilomycin A1 (Figure 4C) and the lysosomal autophagy inhibitor, Lys05 (Figure 4D). These results suggested that pitavastatin-mediated autophagy flux blockade could stabilize FOXO3a. We also found that the stabilized FOXO3a protein by autophagy flux blockade could undergo nuclear translocation upon pitavastatin treatment through confocal microscopy (Figure 4E) and western blot analysis (Figure 4F). Notably, the decrease in cell viability by pitavastatin was alleviated by FOXO3a gene silencing, which strongly suggested that the stabilized FOXO3a was involved in the pitavastatin-mediated apoptotic processes in SCC15 and SW480 cells (Figure 4G).

### Pitavastatin Induces ER Stress Response Leading to Apoptosis

**FIGURE 5:**
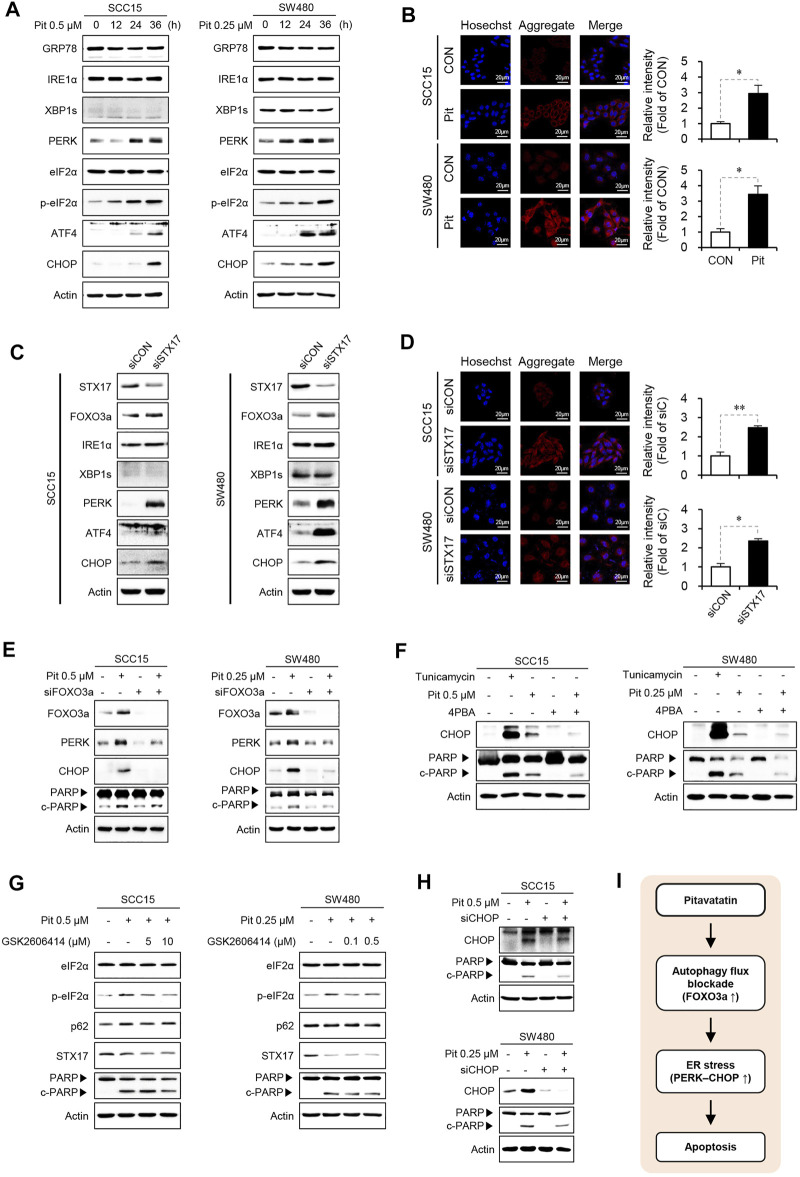
FOXO3a induces ER stress and apoptosis **(A)** SCC15 and SW480 cells were treated with pitavastatin for the indicated time durations. The protein expressions of GRP78, IRE1α, XBP1s, PERK, eIF2α, p-eIF2α, ATF4, and CHOP were detected by western blot analysis, using actin as the loading control. **(B)** SCC15 and SW480 cells were treated with pitavastatin for 24 h, cells were stained with proteostat fluorescent dye, and the formation of protein aggregates was measured by confocal microscopy. Hoechst was used to label the nuclei of cells. The histograms show the quantification performed using the ImageJ software. Statistical analysis was performed using the Student’s *t*-test. Error bars represent mean ± SD (*n* = 3). ^*^ *p* < 0.05. **(C)** SCC15 and SW480 cells were transfected with STX17 siRNA, and the expressions of indicated proteins were measured by western blot analyses. Actin was used as the loading control. **(D)** Cells were transfected with STX17 siRNA, followed by cell staining with proteostat fluorescent dye (stains protein aggregates) and Hoechst (stains nucleus); the protein aggregates were analyzed by confocal microscopy. Statistical analysis was performed using Student’s *t*-test. Error bars represent mean ± SD (*n* = 3). ^*^ *p* < 0.05; ^**^ *p* < 0.01. **(E–H)** Cells were transfected with FOXO3a siRNA in the presence or absence of pitavastatin for 24 h **(E)**, treated with tunicamycin (1 µM) or 4PBA (1 mM) in the presence or absence of pitavastatin for 24 h **(F)**, treated with the indicated concentration of GSK2606414 in the presence or absence of pitavastatin for 24 h **(G)**, or transfected with CHOP siRNA in the presence or absence of pitavastatin for 24 h **(H)**. The expressions of indicated proteins were detected by western blot analyses. **(I)** Proposed model of the anti-cancer effect of pitavastatin.

Recent studies show that statin can cause cell death by inducing UPR and ER stress (Mollazadeh et al., 2018; Dastghaib et al., 2020). These reports suggest that there may be a link between pitavastatin-mediated autophagy and ER stress. Thus, we evaluated the effects of pitavastatin on the UPR arms (IRE1 and PERK) in cells. Pitavastatin time-dependently increased the expression of UPR marker proteins such as PERK, p-eIF2α, ATF4, and CHOP in SCC15 and SW480 cells. However, the expression of GRP78, IRE1α, and XBP1s was not increased by pitavastatin treatment (Figure 5A). Therefore, these results indicate that pitavastatin induces ER stress response which are mainly driven by PERK-CHOP pathway. We next analyzed the formation of protein aggregates by pitavastatin treatment using the proteostat fluorescent dye. The aggresomal formation is a cellular response to the accumulation of misfolded protein as a consequence of ER stress (Wells and Ashizawa, 2011) and the appearance of red signals represent misfolded protein aggregates (Nakajima and Suzuki, 2013; Ohnishi et al., 2013). Our confocal microscopy data showed that pitavastatin could significantly increase the intensity of red signals in the cytoplasm, the indicator of aggresomal formation (Figure 5B). Moreover, the expressions of PERK and CHOP (not IRE1α and XBP1s) (Figure 5C), and the aggresomal formation (Figure 5D) were further enhanced by gene silencing of STX17, which confirm that induction of PERK-CHOP pathway was related to the blockade of autophagy flux. These results proved that pitavastatin-mediated autophagy flux blockade could induce ER stress response. Next, we determined whether FOXO3a stabilized by pitavastatin-mediated autophagy flux blockade could modulate the induction of ER stress. Thus, FOXO3a gene expression was silenced and the expressions of ER stress markers and cleaved PARP protein were evaluated. Our data showed that the increased expressions of PERK and CHOP by pitavastatin were abolished by gene silencing of FOXO3a, which suggested that FOXO3a stabilized by pitavastatin could induce ER stress, thereby leading to cell apoptosis (Figure 5E). Additionally, pretreatment of cells with an inhibitor of induction of ER stress, 4-PBA, reduced the pitavastatin-induced PARP cleavage and CHOP expression (Figure 5F). More specifically, we treated SCC15 and SW480 cells with the selective PERK inhibitor (PERKi), GSK-2606414, or IRE-1α inhibitor, MKC8866, to assess whether these inhibitors were involved in the regulation of pitavastatin effects on apoptosis. As a result, pretreatment of cells with the selective PERK inhibitor GSK2606414 (PERKi) reduced the pitavastatin-induced PARP cleavage (Figure 5G), whereas IRE1α inhibitor MKC8866 did not (Supplementary Figure S1). These results demonstrate that PERK-CHOP signaling might be involved in pitavastatin-induced apoptosis. Consistently, gene silencing of CHOP reduced the PARP cleavage mediated by pitavastatin (Figure 5H). Simultaneously, the possible regulation of autophagy flux by these inhibitors was investigated. We found that inhibition of PERK or IRE1 signaling did not affect the expression of the autophagy flux related proteins, p62 and STX17 (Figure 5G and Supplementary Figure S1). Taken together, these results confirmed that autophagy flux blockade upon pitavastatin treatment was predominantly involved in the induction of apoptosis by modulation of ER stress in both SCC15 and SW480 cells (Figure 5I).

## Discussion

Autophagy is a highly conserved catabolic process by which unnecessary substances or dysfunctional cellular components are either captured and degraded in autophagolysosomes or recycled (Denton and Kumar, 2019). Autophagy acts as a protective mechanism when the cells are encountered by diverse cellular stresses such as nutrient deprivation and hypoxia (Devis-Jauregui et al., 2020). However, autophagy is also known to induce cell death independent of apoptosis, referred to as autophagy-dependent cell death (ADCD) (Bialik et al., 2018; Denton and Kumar, 2019). Altogether these studies indicate the dual nature of autophagy and the possibility that the complete autophagy process can cause cancer cell death or apoptosis. It is believed that excessive autophagosome accumulation due to decreased lysosomal activity is futile for autophagy. This is called incomplete autophagy, and accumulating evidence suggests that incomplete autophagy induction is closely related to apoptosis in several cancers (Vegliante and Ciriolo, 2018; Denton and Kumar, 2019; Lee et al., 2019). The increase in autophagosome synthesis in cell models expressing mutant huntingtin and alpha-synuclein proteins compromise lysosomal activity, while blocking autophagosome synthesis by depleting the autophagy-related proteins, ATG16L and Beclin 1, thereby significantly ameliorating cell death, further suggesting that excessive autophagosome accumulation compromises cell viability (Button et al., 2017). Additionally, the autophagosomal membrane serves as a platform for the induction of caspase-mediated apoptosis where ATG5 and p62 proteins localized on the autophagosomal membrane recruit and bind to the intracellular death-inducing signaling complex (iDISC) for caspase-8 self-processing, further leading to apoptosis induction (Young et al., 2012).

However, in recent years, autophagy-mediated cancer cell death and apoptosis by statins are believed to be due to the impairments in the late stages of the autophagy process, autophagy flux (Tilija Pun et al., 2020). Consistently, lovastatin increases the sensitivity of temozolomide by impairing the autophagosome-lysosome fusion machinery due to the suppression of LAMP2 and dynein, important proteins for the fusion of the autophagosome with the lysosome. The autophagy inducing effect of lovastatin is mediated by the suppression of the Akt/mTOR signaling pathway in the glioblastoma multiforme (GBM) (Zhu et al., 2019). Another statin, mevastatin, in combination with histone deacetylase inhibitors increase its anti-cancer effects by blockade of the autophagy flux mediated by preventing Vps34/Beclin1 complex formation and downregulating prenylated Rab7, a small GTPase necessary for the autophagosome-lysosome fusion (Lin et al., 2017). Ghavami group have recently demonstrated that simvastatin increases temozolomide (TMZ)-induced apoptosis via targeting autophagy flux in GBM cells (Shojaei et al., 2020). This group also reported that simavastatin induces UPR and inhibition of UPR alleviates the effect of simvastatin-TMZ on autophagy flux inhibition (Dastghaib et al., 2020). These findings suggest that inhibition of autophagy flux and subsequent induction of cell death is connected to the IRE1 and PERK signaling arms of the UPR in cell-type dependent manner. Although these sets of evidence differ in part from or to some extent consistent with our findings, many studies are still needed to determine the causes and effects of ER stress and autophagy flux by statins.

In this study, we observed that the anti-cancer effects of pitavastatin were accompanied by the induction of autophagy in oral cancer cells, SCC15, and colon cancer cells, SW480 cells (Figure 2). Further, the pitavastatin-mediated apoptosis were confirmed to be due to the blockade of autophagy flux (Figure 3). Different explanations have been put forth to explain the role of autophagy flux blockade in the induction of cell death and apoptosis. For example, blockade of autophagy flux leads to the accumulation of the death receptor 5 (DR5), which in turn induces apoptosis upon TRAIL treatment in lung cancer cells (Zinnah and Park, 2019). Previous studies also show that the autophagy flux blockade-mediated accumulation of p62 is the trigger protein that leads to caspase-9- or caspase-8-mediated apoptosis induction (Young et al., 2012; Feng et al., 2018). However, the accumulated p62 did not exert an apoptotic role in our experimental conditions (data not shown). In the present study, an additional key molecule that plays a role in the induction of apoptosis following autophagy flux blockade in pitavastatin-treated conditions was identified. As FOXO3a protein undergoes turnover through the autophagy process (Fitzwalter and Thorburn, 2018; Fitzwalter et al., 2018), we confirmed the expression of the FOXO3a protein under the conditions where the autophagy flux was blocked. Interestingly, we found that all these conditions of autophagy flux blockade, including treatment with bafilomycin A1 (Figure 4C), lysosome inhibitor, Lys05 (Figure 4D), and gene silencing for STX17 (Figure 5C) led to a significant increase in the expression of FOXO3a protein. Moreover, this increased expression of FOXO3 was not due to changes at the gene level (data not shown), but to an increase in protein stability (Figure 4B). We also found that the decreased cell viability by pitavastatin in cells was partially recovered upon FOXO3a gene silencing (Figure 4G). This result was consistent with the recent finding, wherein the accumulation of FOXO3a protein by the blockade of autophagy flux causes mitochondrial apoptosis in non-small-cell-lung carcinoma (Guo et al., 2019).

In our previous study, we observed that FOXO3a induced apoptosis by increasing PUMA expression in oral squamous cancer cells (Lee et al., 2020). However, in the present study, we were able to explore a different role of FOXO3a in the induction of apoptosis in SCC15 and SW480 cells through the induction of ER stress response, particularly by stimulating PERK-CHOP axis (Figure 5). In particular, gene silencing of FOXO3a decreased the expression of total PERK and CHOP levels along with the expression of c-PARP, which suggested that FOXO3a regulated ER stress (Figure 5E). Moreover, pharmacological inhibition of ER stress by 4PBA and PERKi, and (or) gene silencing of CHOP, could significantly reduce cleaved-PARP expression by pitavastatin (Figures 5F–H). However, how FOXO3a was able to escape autophagosomes to induce transcription and ER stress remains unknown. A previous study shows that FOXO3a induces PERK expression by directly regulating its transcriptional induction (Alasiri et al., 2019), which supports our findings that the blockade of autophagy flux causes the accumulation of FOXO3a, thereby leading to induction of CHOP, and subsequently leading to apoptosis. Another possibility also may exist. Reactive oxygen species (ROS) have recently emerged as important regulators of ER function and leads to ER stress-induced cell death (Verfaillie et al., 2012). In this context, recent study has shown that FOXO3a-dependent ROS production could induce cell death by apoptosis and ER stress in cerebral ischemia (Shi et al., 2019). This suggests the possibility that ROS could be preemptively involved in ER stress induced by FOXO3a. In further studies, we need to investigate a close link between activation of FOXO3a and ROS to identify a detailed mechanism for how FOXO3 causes ER stress.

Further, the inhibition of autophagy flux by knockdown of STX17 increased PERK and CHOP expression which was consistent with the accumulation of FOXO3a (Figure 5C). Consistent with our results, other studies also show that impairment in autophagy flux is associated with the induction of ER stress, thereby leading to the induction of apoptosis (González-Rodríguez et al., 2014). Blockade of autophagy flux can ubiquitinate protein aggregates leading to ER stress, CHOP induction, and apoptosis (Moriya et al., 2013). Induction of ER stress by the blockade of autophagy flux is ameliorated by the overexpression of STX17 which resumes autophagy flux to provide protection against ischemic neuronal injury (Chen et al., 2020). The knockdown of STX17 aggravates ER stress-dependent neuronal apoptosis induced by ischemic/reperfusion (Chen et al., 2020), and this result was consistent with the present data which showed that gene silencing of STX17 could enhance the expression of ER stress markers, PERK, and CHOP (Figure 5C). In contrast to our study, previous research highlights an opposite pathway, wherein ER stress can inhibit autophagy flux, most likely by inhibiting the lysosomal activity (González-Rodríguez et al., 2014). Cathepsin B is a lysosomal cathepsin involved in nonspecific protein degradation in lysosomes. Inhibition of cathepsin B leads to massive accumulation of non-fused autophagosomes (Rudzinska et al., 2019). The decrease in cathepsin B and cathepsin D activities without any effects on autophagosome-lysosome fusion increases the cytotoxicity of cisplatin and paclitaxel in the lung cancer cells (Wang et al., 2020). Thus, the decrease in cathepsin B activity by pitavastatin in the present study (Figure 3A) may also affect apoptosis, although the direct effect of cathepsin B in cell viability and apoptosis was not investigated.

In summary, present study showed that pitavastatin induces apoptosis in SCC15 and SW480 cells by inhibiting autophagy flux and inducing UPR. Pitavastatin-induced impairment of autophagy flux and subsequent induction of apoptosis is mainly associated with activation of PERK-CHOP pathway. Taken together, this study shows the potential anti-cancer effects of pitavastatin and targeting autophagy flux could be a promising strategy for the therapeutic interventions in cancer treatment.

## Data Availability

The original contributions presented in the study are included in the article/Supplementary Material, further inquiries can be directed to the corresponding author.
