# Supplementary figures and images for: Pitavastatin Induces Cancer Cell Apoptosis by Blocking Autophagy Flux
Source: Front Pharmacol. 2022 Mar 21;13:854506. doi: 10.3389/fphar.2022.854506 (PMC8977529; doi:10.3389/fphar.2022.854506)

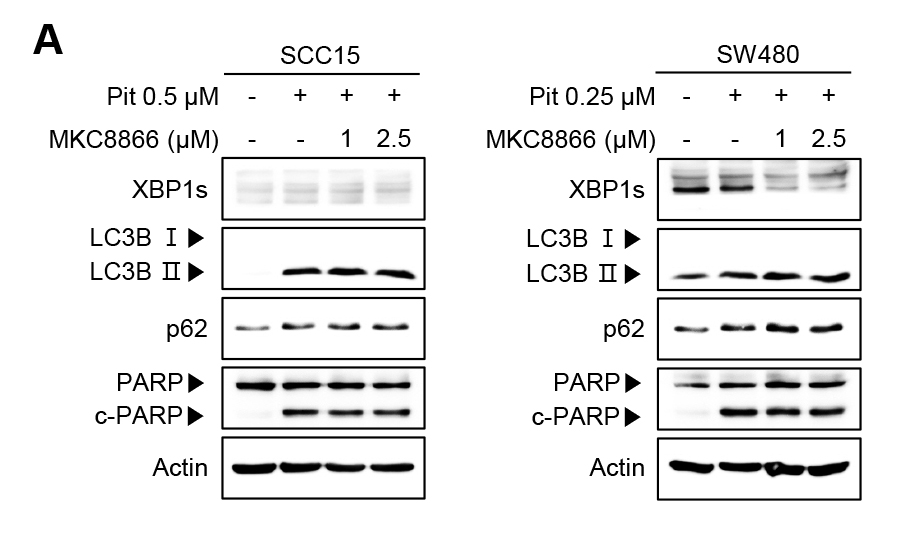

Supplement: Supplementary file 1 [file Image1.JPEG]
